# Epidemiological profile of acute Chagas disease in individuals infected by oral transmission in northern Brazil

**DOI:** 10.1590/0037-8682-0088-2020

**Published:** 2020-09-11

**Authors:** George Harisson Felinto Sampaio, Andressa Noronha Barbosa da Silva, Carlos Ramon do Nascimento Brito, Nathan Ravi Medeiros Honorato, Luara Musse de Oliveira, Antônia Claudia Jácome da Câmara, Lúcia Maria da Cunha Galvão

**Affiliations:** 1 Universidade Federal do Rio Grande do Norte, Centro de Ciências da Saúde, Programa de Pós-Graduação em Ciências da Saúde, Natal, RN, Brasil.; 2 Universidade Federal do Rio Grande do Norte, Centro de Ciências da Saúde, Programa de Pós-Graduação em Ciências Farmacêuticas, Natal, RN, Brasil.; 3 Universidade Federal do Rio Grande do Norte, Centro de Ciências da Saúde, Departamento de Análises Clínicas e Toxicológicas, Natal, RN, Brasil.; 4 Universidade Federal do Rio Grande do Norte, Centro de Biociências, Programa de Pós-Graduação em Biologia Parasitária, Natal, RN, Brasil.; 5 Instituto Federal do Pará, Breves, PA, Brasil.

**Keywords:** Trypanosoma cruzi, Açaí, Acute infection

## Abstract

**INTRODUCTION:**

Oral infection by *Trypanosoma cruzi* is currently the most important route of transmission of acute Chagas disease (ACD) in the North region of Brazil, and the reported outbreaks are usually related to ingestion of contaminated food, especially unprocessed *açaí* pulp.

**METHODS:**

A retrospective cohort study was performed to analyze the epidemiological profile of individuals with suspected cases of ACD in the municipality of Breves, located in the state of Pará, Brazil. Therefore, notifications of suspected cases of ACD were collected from the Municipal Health Department of Breves from January 2007 to December 2017.

**RESULTS:**

A total of 265 individuals were registered, and the majority were male (54.7%; 145/265). Age ranged from nine months to 79 years, with a greater number of notifications for individuals aged between 1 and 39 years (71.3%; 189/265). Most of them had a low level of education (74.3%, 197/265), were living in rural and urban areas (58.9%; 156/265 and 37.7%; 100/265, respectively). Infection occurred mainly in the domestic environment (96.2%; 255/265) through oral transmission (98.1%; 260/265). There were a greater number of notifications in November, December and January.

**CONCLUSIONS:**

These data showed that oral transmission of *T. cruzi* has become increasingly high in the study region, and health education programs need to be implemented as strategies to ensure good manufacturing practices of unprocessed food.

## INTRODUCTION

American trypanosomiasis or Chagas disease, an anthropozoonosis caused by *Trypanosoma cruzi*, is one of the 17 neglected tropical diseases that still persist in the poorest societies and affect approximately six million people worldwide^1^. Transmission to human beings can occur in several ways: vectorial, blood transfusion, congenital including oral transmission, and also through transplants of organs from infected people, laboratory accidents and sexual intercourse^2^.

Vector transmission, which happens only in the endemic countries of Latin America, was considered as the main transmission route of the parasite for several decades^3^. Currently, as a result of larger migration flows, chagasic infection is no longer found only in endemic areas of Latin America; it is now also diagnosed in countries considered to be non-endemic. Thus, *T. cruzi* infection has become a major global health problem, with treatment deficiencies and absence of effective vaccines^4^. However, transmission remains active in some ecologically viable regions. The main problems include poor maintenance of vector surveillance, new epidemiological situations, oral transmission, wild and secondary vectors, resistance to insecticides, urbanization and migration of infected individuals, restrictive policies such as instability, decentralization of health programs, and millions of existing cases requiring medical attention^5^.

Oral transmission of *T. cruzi* to humans was first reported in Brazil in 1967 in the municipality of Teutônia, state of Rio Grande do Sul^6^. From 1968 to 2000, 50% of acute cases of Chagas disease in the Amazon region were attributed to oral transmission^7^, and between 2000 and 2010, the rate reached 70%^8^. This route of transmission is frequent in the Brazilian Amazon region^9^ ^-^ ^11^, and it is responsible for the two largest outbreaks of acute Chagas disease reported to date^12^ ^,^ ^13^. Outbreaks of orally-transmitted *T. cruzi* infection have been reported in several Latin American countries, such as Colombia, Venezuela, Bolivia and French Guiana^14^. For most of the outbreaks that have been stated, the epidemiological profile indicates non-vector transmission, involving intake of juice made of local fruits^15^. 

One of the main suspected sources of *T. cruzi* infection is the ingestion of tainted food, such as *Euterpe oleracea* Mart. (*açaí*), which is widely consumed as a drink made from a mixed pulp^16^. However, this relevant transmission pathway is underestimated and understudied^17^. In Brazil, 24 cases of acute human Chagas disease were detected in Santa Catarina State, and all of them were related to ingestion of *T. cruzi*-contaminated sugarcane juice^18^. Recently, another outbreak of ACD resulting from infection by sugarcane juice, occurred in the city of Marcelino Vieira, state of Rio Grande do Norte (RN), with 18 confirmed cases^19^. Another outbreak was reported in the municipality of Ibimirim, in the state of Pernambuco, where 27 people were positive for *T. cruzi* after attending the same religious event. The source of contamination is still under review, but evidence points to raw foods^20^. In the period between 2012 and 2016, 1,190 cases of ACD were confirmed and 18 deaths were recorded, with an annual mortality rate of 1.5%. Most of the cases (82%) occurred in the North region, and 12% of the municipalities in this region registered cases of ACD in five consecutive years; nine municipalities are located in the state of Pará and one, in the state of Amapá. Approximately 52% of individuals with ACD live in urban areas^17^. Recent data collected in the Brazilian Amazon have described the occurrence of an outbreak of acute Chagas disease (ACD) involving ten patients whose suspected source of contamination was the ingestion of *açaí* pulp. Analysis of the patients’ blood samples and the juice samples contained *T. cruzi*, genotyped as TcIV, indicating oral transmission of the etiologic agent of the infection^16^. It should be noted that many cases of ACD may remain unnoticed, mainly as a result of the difficulty of diagnosis in the acute phase, which is usually limited by the lack of commercial kits registered with the National Health Surveillance Agency (ANVISA), and also because of clinical symptoms that are nonspecific to Chagas’ disease^21^. 

In this context, the present study evaluates the epidemiological profile of individuals suspected of ACD in the municipality of Breves, State of Pará, Brazil, and to understand the route of infection in order to help improve public policies to prevent the oral transmission of *T. cruzi*. 

## METHODS

### Study area

**FIGURE 1:**
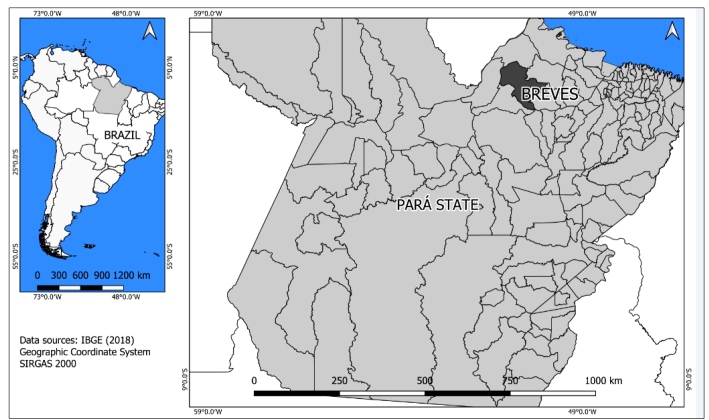
Map of Brazil highlighting the state of Pará and the municipality of Breves (in black).

The study was carried out in the municipality of Breves, in the north of the state of Pará, in northern Brazil. The municipality is located in the southwest of Marajó Island, at latitude 01º40'56” South and longitude 50º28'49" West (Figure 1). Breves has a humid equatorial climate with high temperatures and high rainfall, with an area of approximately 9,566 km^2^. The population was estimated at 92,860 inhabitants in 2010. The local economy is based mainly on extractivism, especially extraction of *açaí*, in addition to fishing, mining, logging, production of Brazil nuts, as well as family farming and tourism^15^.

### Study design

This is a retrospective cohort study, using secondary data provided by the Municipal Health Department of Breves, for the period from January 2007 to December 2017. All of the study informants lived in the municipality of Breves and were treated at the municipal hospital. Eight demographic variables were assessed: month of onset of clinical symptoms, age, education, sex, type of dwelling, form of transmission, place of infection and laboratory-confirmed diagnosis. Suspected cases were attributed to individuals who had clinical manifestations associated with ACD, as cases of the disease had been detected in the region, and the people infected had reported intake of fresh *açaí* (artisanal production).

Individuals were considered to be positive in laboratory tests when the parasite was detected by the direct parasitological method, based on the detection of trypomastigote forms in the blood. Samples from the subjects were considered to be negative in the absence of trypomastigotes during direct examination of peripheral blood. Data were analyzed using descriptive statistics, and the results were presented in tables and graphs as absolute and relative frequencies.

## RESULTS

A total of 265 individuals suspected of *T. cruzi* infection were investigated in the municipality of Breves, in the state of Pará, Brazil, from 2007 to 2017. The socio-demographic profile of these individuals showed that the majority were male (54.7%). The subjects’ age ranged from nine months to 79 years, with a greater number of cases reported between 1-19 years (38.1%) and 20-39 years of age (33.2%). They had a low level of education, and 71.7% of them had not completed high school. Individuals lived in rural (58.9%), urban (37.7%) and peri-urban areas (2.3%) (Table 1).

**TABLE 1:** Epidemiological variables of individuals notified with suspected acute Chagas disease in the municipality of Breves, Pará State, Brazil from 2007 to 2017.

Epidemiological variables		Number	Percentage
Gender	Male	145	54.7
	Female	120	45.3
	**Total**	**265**	**100**
Age range	< 1 year old	3	1.1
	1-19 years old	101	38.1
	20-39 years old	88	33.2
	40-59 years old	45	17.0
	> 60 years old	28	10.6
	**Total**	**265**	**100**
Education level	Illiterate	29	10.9
	Middle/Elementary school	161	60.8
	High school	29	10.9
	Higher Education	6	2.3
	Not applicable	24	9.1
	Ignored/blank	16	6.0
	**Total**	**265**	**100**
Area of residence	Urban	100	37.7
	Rural	156	58.9
	Peri-urban	6	2.3
	Ignored/blank	3	1.1
	**Total**	**265**	**100**

**FIGURE 2:**
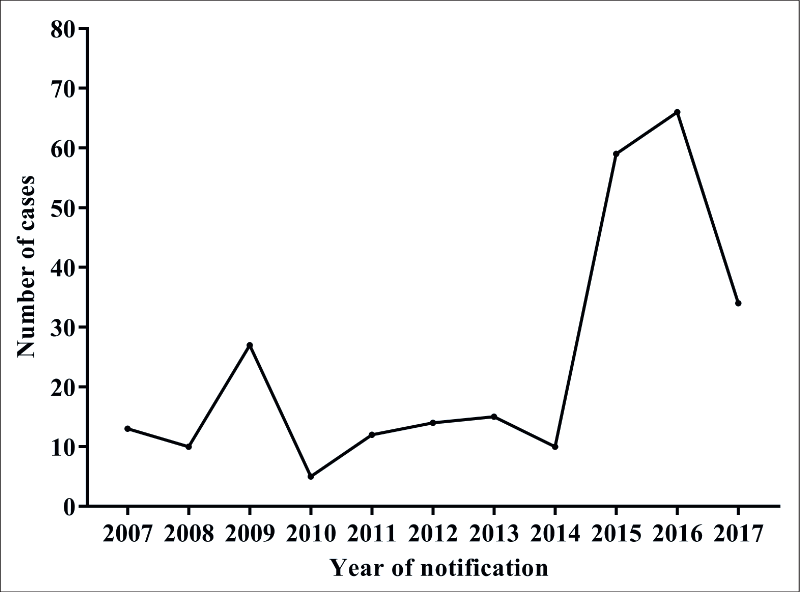
Annual distribution of suspected cases of acute Chagas disease among inhabitants of the municipality of Breves, state of Pará, Brazil, from 2007 to 2017.

**FIGURE 3:**
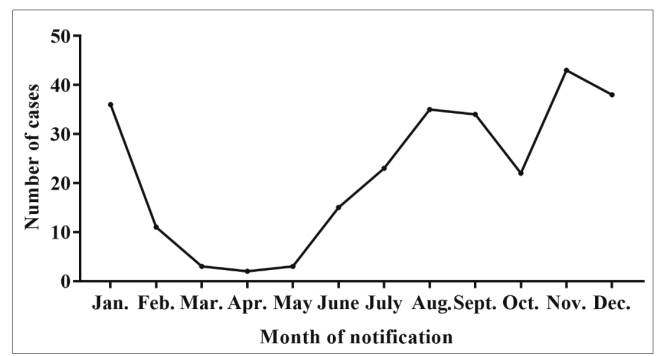
Distribution of suspected cases of acute Chagas disease among inhabitants of the municipality of Breves, state of Pará, Brazil, for the month of onset of symptoms in the period from 2007 to 2017.

There were variations in the number of cases notified between 2007 and 2014, and there was a progressive increase from 2014 to 2016 (Figure 2). Most of the individuals were infected in their household (96.2%, 255/265), and the main infection route was oral transmission of the parasite (98.1%; 260/265). The notifications were reported mainly in the months of November, December and January (Figure 3), suggesting that ACD by oral infection has a seasonal profile in this region. Laboratory diagnosis was confirmed in 64.2% (170/265) of the cases while 27.5% (73) were negative (Table 2).

**TABLE 2:** Number of individuals notified with suspicion of acute Chagas disease, route and place of transmission, and laboratory tests in the city of Breves, Pará State, Brazil, from 2007 to 2017.

Epidemiological variebles		Number	Percentage
Route of transmission	Vectorial	4	1.5
	Oral	260	98.1
	Ignored/blank	1	0.4
	**Total**	**265**	**100**
Place of transmission	Domicile	255	96.2
	Other	10	3.8
	**Total**	**265**	**100**
Laboratory tests	Positive	170	64.2
	Negative	73	27.5
	Unperformed	18	6.8
	Ignored/blank	4	1.5
	**Total**	**265**	**100**

## DISCUSSION

The epidemiological profile of suspected cases of acute Chagas’ disease by oral transmission showed that *T. cruzi* infection occurred mainly in male individuals whose age ranged from nine months to 39 years old; they had incomplete high school education, and lived in the rural area. Epidemiological data of the state of Pará, collected from the DATASUS database from 2006 to 2012, confirmed a profile for the region with a high percentage of oral transmission (68.4%) in a total of 977 patients diagnosed with ACD^22^. A recent study using data from the Notifiable Diseases Information System (SINAN) - Ministry of Health, showed epidemiological profile similar to the present study: 69% of respondents were aged between 18 and 60 years, and 38% were male and 31% female. These respondents represent the group most affected by oral transmission of *T. cruzi* in the region^22^. However, we cannot ignore the fact that human-vector interactions are possible even with low frequency of triatomines indoors^11^. The municipality of Breves is located in northern Brazil, where non-domiciled triatomines are sporadically found indoors. Most cases of *T. cruzi* infection have been attributed to oral transmission and a few have been reported^16^.

The epidemiological scenario of Chagas disease in Breves showed that the oral route is the main form of transmission of *T. cruzi*, corroborating recent^22^ and old^23^ data, and occurs mainly in households (96.2%). In the present study, there was a high rate of *T. cruzi* oral transmission, and it has become increasingly frequent in this region, when compared to previous research. In other regions, where oral transmission by *T. cruzi* is less frequent, for example, in the state of Pernambuco, this route of transmission has significantly decreased after 2006, a period in which Brazil received an international certificate for interrupting vector transmission *by Triatoma infestans* ^24^. 

The data provided for this study by the Municipal Health Department did not allow a description of the clinical manifestations present in individuals with suspected oral transmission of the parasite. However, a recent analysis of records from the SINAN in the state of Pará showed that the most frequent clinical manifestations in orally-infected individuals were fever, asthenia, facial or lower limb edema and generalized edema^22^.

The progressive increase in notifications of suspected ACD cases associated with oral transmission by *T. cruzi*, as found in this study, follows the same profile reported in Brazil: between 1968 and 2005, 437 cases of acute Chagas disease were reported; 311 of them were linked to 62 outbreaks caused by ingestion of *açaí* pulp. In contrast, from 2000 to 2010, there were more than 1,000 acute cases in 138 outbreaks, with 776 cases (71%) attributed to orally-transmitted epidemics^15^. The number of ACD notifications was found to be period-specific, and was higher in the months of November, December and January. This finding suggests that seasonal infection by *T. cruzi* is associated mainly with oral transmission. The period corresponding to the peak of ACD notifications coincides with the *açaí* harvest months in this region. This finding reinforces the relationship between this food and oral transmission of *T. cruzi*. However, other factors may contribute to increased infection by this parasite; for example, longer life expectancy and the growing number of *açaí* consuming markets without certification for parasitological quality^25^ ^-^ ^27^. Such factors were identified as potential sources of transmission of the parasite and, consequently, of ACD, which could become an important public health problem in the coming decades.

Historically, micro-epidemics of community-based oral infection of ACD are responsible for human cases with severe symptoms that can be followed by death^28^ ^-^ ^30^. *T. cruzi* oral transmission generally coincides with warm climates, which causes triatomines to remain more active, with greater mobility and hematophagy. As a consequence, there is a stronger possibility of contamination of food and the environment with feces and/or urine containing metacyclic trypomastigote forms. Depending on temperature and humidity levels, *T. cruzi* can stay alive for a few hours or even for days; at low temperatures, its viability can last for weeks. Thus, contaminated food kept moist and in a liquid or pasty state favors resistance and, consequently, transmission of the parasite. The *açaí* is an ideal source for transmission of the parasite, because its fruits are grown, harvested and manipulated with the help of artificial light for artisanal preparation in rural or peri-urban areas, where triatomines are often abundant and there is no sanitary control^25^ ^,^ ^31^. However, the parasite can be neutralized by heating at 43ºC for 20 min^32^.

Prevention and control measures must be adopted at all stages of the *açaí* production chain, both by large producers that do perform pasteurization and by artisan processors. In the state of Pará, hygienic-sanitary procedures have been established for the handling of *açaí* by artisan processors since 2012. These procedures are a way of reducing the initial microbial load, minimizing risks, and preventing outbreaks of foodborne diseases. The process consists of three washes of the fruits with drinking water; the second wash is performed with hypochlorite at the concentration of 150 PPM of active chlorine. Then, the bleaching step should be performed by immersing *açaí* in heated water at 80ºC for 10 s and then in cold water. Best practices have been introduced in the entire *açaí* production chain to improve the hygienic-sanitary conditions of the processing units, offering consumers a safe product, in addition to promoting public policies for social inclusion in this segment of the production chain^33^.

The State of Pará is the largest producer and consumer of *açaí* worldwide. This fruit is traditionally consumed unprocessed and is the basis of the cuisine of Pará; it is one of the most important supplements in the Brazilian diet of North region^34^. It is worth mentioning that irrigation of land for cultivation of *açaí* is a common practice throughout year in the municipality of Breves. This practice results in high availability of the fruit, which may increase the risk of infection with *T. cruzi* in this region. In these cases, ACD outbreaks have a high socioeconomic impact in the North of Brazil^35^.

Of the 265 individuals suspected of ACD, 73 (27.5%) tested negative in a parasitological examination of peripheral blood for *T. cruzi* infection. The parasitological diagnosis of Chagas disease in the acute phase is based on the detection of the parasite, and test sensitivity depends on the number of parasites in the peripheral blood, which is usually high. For suspected cases of acute infection, different direct parasitological methods can be used for immediate and repeated reading to clarify the diagnosis^36^ ^-^ ^38^. These methods are: Strout concentration, micro-hematocrit test and buffy coat, and they are recommended as the first choice for symptomatic diagnosis of cases with more than 30 days’ evolution, because of the decline in parasitemia over time^39^. Finding negative results for the parasite in direct examination of peripheral blood suggests the need to use a serological test to detect anti-*T.cruzi* IgM antibodies. This test indicates a positive acute phase, especially when combined with epidemiological features and clinical manifestations^40^. Difficulties in the diagnosis of the acute phase of ACD can be explained by the lack of commercial kits registered in the National Health Surveillance Agency for obtaining positive controls for IgM^36^, as well as by the possibility of false positive results in several febrile illnesses^21^. 

The limitation of this study refers to the lack of other data available in the database, e.g., reported clinical manifestation, individuals’ knowledge of triatomines, risk of oral infection by *T. cruzi* and hygiene measures that should be adopted before consumption of *açaí* fruits. The scope is intended for analysis of data from one municipality only, which represents a significant reduction in the sampling population. Thus, we suggest that the method used in the present study should be employed in the analysis of others municipalities in the northern region of Brazil. Underreporting and the fact that the study is retrospective are relevant limitations. 

Our results demonstrated that the rate of oral transmission of *T. cruzi* through tainted food has become increasingly high in the study region over the years. Therefore, we recommend that high-quality basic education - mainly in the *açaí* harvest months, based on effective public policies - should be extended to the entire state of Pará, including islands far away from the metropolitan region. Such education could be used as a model for the entire North region. This can facilitate inspection to ensure continuous control of efficient hygiene practices in the *açaí* production chain by the population and by artisanal producers. Other measures, e.g., training of health professionals, prevention measures, early diagnosis in case of epidemiological risk (even if far from vector-transmission areas) are also extremely important. We also emphasize the proper care of people infected with *T. cruzi* as an essential strategy for a comprehensive control of Chagas infection and its eradication as a public health problem^1^ ^,^ ^31^. These findings call for better hygiene control in fresh food handling, for artisanal *açaí* in particular, in order to avoid infection by *T. cruzi* and to increase the safety and viability of consumption of this fruit, which is a great nutritional source and has high economic importance for the northern region of Brazil.
